# Expression of Genes Involved in Anthracnose Resistance in Chili (*Capsicum baccatum*) ‘PBC80’-Derived Recombinant Inbred Lines

**DOI:** 10.3390/pathogens12111306

**Published:** 2023-11-01

**Authors:** Wassana Kethom, Paul W. J. Taylor, Orarat Mongkolporn

**Affiliations:** 1 Department of Horticulture, Faculty of Agriculture at Kamphaeng Saen, Kasetsart University, Kamphaeng Saen Campus, Nakhon Pathom 73140, Thailand; wassana.kethom@gmail.com; 2 Faculty of Science, The University of Melbourne, Parkville, VIC 3010, Australia; paulwjt@unimelb.edu.au

**Keywords:** *Colletotrichum scovillei*, hypersensitive reaction, microinjection, wounded inoculation, defense-related genes, qRT-PCR

## Abstract

Chili anthracnose has long been a threat to chili production worldwide. *Capsicum baccatum* ‘PBC80’ has been identified as a source of resistance to anthracnose. Recently, a QTL for ripe fruit resistance from ‘PBC80’-derived RILs was located on chromosome 4 (123 Mb) and contained over 80 defense-related genes. To identify the genes most related to anthracnose resistance, a fine map of the QTL region was developed using single-marker analysis. Nine genes were selected from the new QTL (1.12 Mb) to study their expression after being challenged with *Colletotrichum scovillei* ‘MJ5’ in two different RIL genotypes (Resistance/Resistance or R/R and Susceptible/Susceptible or S/S) at 0, 6 and 12 h. Of the nine genes, *LYM2*, *CQW23_09597*, *CLF*, *NFXL1*, and *PR-14* were significantly up-regulated, compared to the control, in the R/R genotype. *ERF* was up-regulated in both chili genotypes. However, the expression was relatively and constantly low in the S/S genotype. Most up-regulated genes reached the highest peak (2.3–4.5 fold) at 6 h, except for *ERF*, which had the highest peak at 12 h (6.4 fold). The earliest and highest expressed gene was a pathogen receptor, *LYM2*.

## 1. Introduction

Chili (*Capsicum* spp.) is an economically important global crop as a key vegetable and spice [1]. A complex of *Colletotrichum* species causes chili anthracnose. In Thailand, the most important species are *Co. scovillei*, *Co. truncatum*, and *Co. siamense* [2], with *Co. scovillei* being the most aggressive species [3]. Among the cultivated *Capsicum* species, *C. annuum* lacks resistance to anthracnose, while *C. baccatum* has resistance to the pathogen, especially *Co. scovillei*. *Capsicum baccatum* ‘PBC80’ accession has been used widely in Asia and Thailand as the resistant source in several chili-breeding programs [1].

Disease resistance in crop plants is generally controlled by a few genes that have large effects on the phenotype; however, quantitative variations are also observed [4,5]. Genetic studies for anthracnose resistance in chili with the hypersensitive reaction (HR) have mostly shown single genes controlling the resistance [6,7,8,9], but some variations were detected in the susceptible phenotypes. Such variations suggested that the resistance to anthracnose in chili was a quantitative trait. The resistance trait’s quantitative trait locus (QTL) refers to the trait’s genomic region, which is identified by a statistical QTL analysis aiming to link phenotype and genotype and explain the genetic basis of variation in the traits [4,5,10].

Mapping and QTL analyses of the resistance genes to anthracnose derived from the ‘PBC80’ have been achieved [9,11]. The most recent map developed by Kethom and Mongkolporn [11] was derived from the recombinant inbred lines (RILs) developed from an intraspecific *C. baccatum* cross ‘PBC80’ × ‘CA1316’. The resistance to anthracnose on ripe fruit was located on chromosome 4 within the QTL *RA80f6_r1*. The QTL *RA80f6_r1* region is physically 123 Mb (22,920,913–146,776,687 bp), housing approximately 85 defense-related genes.

Pathogenesis-related proteins are expressed by the host at various time points during infection and are correlated with differential pathogenesis-related genes expressed by the pathogen. The crucial interactions between the fungal pathogen and the host plant occur as early as at the penetration stage of the cuticle and through to the infection/colonization stage. The resistance mechanism in host, *C. baccatum* ‘PBC80’, has been well defined as a hypersensitive reaction (HR) [8]. HR is often a consequence of the plant host recognition of the invasive pathogen, which results in rapid cell death around the infection site [12]. Pathogen recognition by cell-surface pattern recognition receptors (PRRs) is the earliest response event of a plant host that initially induces defense mechanisms to inhibit the pathogen invasion. Studies in rice [13] and coffee [14] revealed that plants could promptly recognize fungal infection and activate defense activities through the early expressions of the PRRs before a full development of fungal appressorium around 5–6 h after inoculation. In chili anthracnose, the causal pathogen *Colletotrichum* was demonstrated to produce appressoria as early as 6 h after inoculation [15]. Since the recognition receptors appeared to be the key genes to activate the HR response, the early response genes were consequently the targets of this study. The study was then planned to collect mRNA at an early stage of the infection within the first 24 h after inoculation.

This study aimed to identify genes involved in the QTL *RA80f6_r1* for resistance to anthracnose from ‘PBC80’ through their mRNA expression post-inoculation. The QTL *RA80f6_r1* was physically large and contained over 80 defense-related genes in the region. Therefore, this study tried to narrow the QTL region by incorporating more markers into the QTL area to achieve a fine map. As a result, the new QTL was physically smaller to accommodate searching targeted defense-related genes, which then had their expressions post-fruit inoculation investigated. 

## 2. Materials and Methods

**Figure 1:**
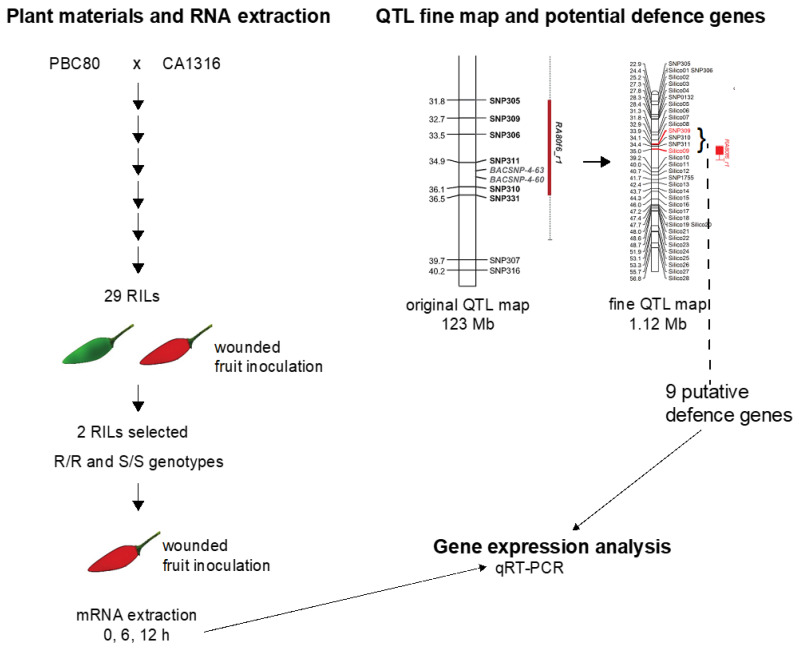
A graphical summary of the study methodology.

A graphical figure to explain the overall study methodology is exhibited in Figure 1.

### 2.1. Fine Mapping in the QTL Region and Selection of Defense-Related Genes

The QTL *RA80f6_r1* previously identified by Kethom and Mongkolporn [11] was derived from homozygous recombinant inbred lines developed from a cross between *C. baccatum* ‘PBC80’ and ‘CA1316’. The QTL was located on chromosome 4 and contained eight molecular markers with a total physical distance of 123 Mb. A fine map of the QTL was attempted by adding more markers into the region. These additional markers were recruited from silico (insertion-deletion mutants) and SNPs (single nucleotide polymorphisms) within the DArTseq [16] genome database of the RIL population [11] by lowering the marker filtering criteria with ≥75% call rate and minimum allele frequency ≥ 0.1. All the recruited markers had known locations on the *Capsicum* chromosome.

The fine mapped QTL was then analyzed to identify markers closely linked to the resistance to anthracnose with single-marker analysis using QTL IciMapping 4.1 software (http://www.isbreeding.net (accessed on 16 August 2022); [17]). The quantitative phenotypic data derived from Kethom and Mongkolporn [11] were converted to binary data as 0 = resistance and 1 = susceptibility. Single-marker analysis based on a simple linear regression [10] was used to identify markers that were most linked to the resistance to anthracnose by showing the highest LOD scores. 

Putative genes involved in the plant defense mechanisms were searched within 7 Mb up- and down-stream of the fine QTL region, and were selected for the gene expression study.

### 2.2. Selection of RILs with Resistance and Susceptibility to Anthracnose

Thirty-one RILs from a cross of *C. baccatum* ‘PBC80’ × ‘CA1316’ were grown in 30 cm plastic pots. Each line contained 1–2 plants. The chili plants were laid in a 32-mesh insect-proof house with 50 × 100 cm spacing, at the Tropical Vegetable Research and Development Center, Department of Horticulture, Faculty of Agriculture at Kamphaeng Saen, Kasetsart University, Nakhon Pathom, Thailand. 

A fruit bioassay was performed to evaluate resistance to anthracnose. Three fruits each at mature green and ripe maturity stages were collected for inoculation. Each fruit was wounded and inoculated with *Colletotrichum scovillei* isolate MJ5 using a microinjector [10]. Anthracnose symptoms were evaluated at 9 days after inoculation. The anthracnose severity was evaluated based on a 0–9 disease score developed by [18], whereby the lesion size proportional to the fruit size was considered as follows: 0 = no infection, 1 = 1–2%, 3 = >2–5%, 5 = >5–15%, 7 = >15–25%, 9 = >25%.

Two RILs, anthracnose-resistance (R/R) and -susceptible (S/S) at both fruit stages, were selected.

### 2.3. Expression Study of the Putative Defense-Related Genes

#### 2.3.1. Fruit Inoculation

The isolate MJ5 was cultured on potato dextrose agar (PDA; Difco, Becton, Dickinson and Company, Sparks, MD, USA) under near-UV for 7 days. When the fungus sporulated, sterilized water was added to the top of the culture and the culture surface was scraped to collect spores. The collected spores were filtered with sterilized muslin cloth. The spores were adjusted to a concentration of 1.0 × 10^6^ spores/mL [18]. 

Fifteen ripe fruit were collected from each chili plant. Calyces were removed and fruit were surface sterilized in 1% (*w*/*v*) sodium hypochlorite solution for 5 min, and then rinsed twice with reverse osmosis water. The clean fruit were laid on a metal sieve in a plastic box half-filled with water. Of the 15 fruit, 9 were inoculated with sterilized water, and 6 were inoculated with the MJ5. Each fruit was injected twice in the middle of the pericarp. The injection wounds were 3 cm apart. Each injection contained 1 μL of either water or spore suspension. The plastic inoculation box lid was closed to maintain high humidity for 12 h.

#### 2.3.2. RNA Extraction and cDNA Synthesis

Total RNA was extracted from the challenged fruit with either water or the MJ5 at 0 (only water inoculation was performed), 6, and 12 h after inoculation, using a modified CTAB-LiCl method [19]. The 1 cm^2^ fruit tissue surrounding the inoculation wound was excised and immediately dipped into liquid nitrogen. The frozen tissue was stored at −80 °C until required for RNA extraction. The quantity and quality of the extracted total RNA was measured with a Nanodrop 2000c spectrophotometer (Thermo Fischer Scientific, Waltham, MA, USA). The RNA was stored at −80 °C.

Before cDNA synthesis, the RNA samples were DNA cleaned by incubating the RNA with DNAse I, RNAse-free (New England Biolabs, Ipswich, MA, USA) at 37 °C for 40 min. One µg of the RNA was converted to cDNA with the RevertAid RT Reverse Transcription Kit (Thermo Fischer Scientific, Waltham, MA, USA). The cDNA quantity and quality were inspected using the Nanodrop 2000c spectrophotometer. The cDNA was diluted 15-fold with RNase-free water, and then transferred to −80 °C storage.

#### 2.3.3. Designing the Primers Specific to the Targeted Defense-Related Genes

The nucleotide data of the selected putative defense-related genes were derived from the *Capsicum baccatum* ‘PBC81’ reference genome, GenBank database “www.ncbi.nlm.nih.gov (accessed on 15 August 2022)”. The secondary structure of each gene was inspected with mfold “http://www.unafold.org (accessed on 16 August 2022)”. The primers specific to the selected genes were designed with Primer3Plus “https://www.primer3plus.com (accessed on 16 August 2022)” (Table 1) by avoiding any expected secondary structure regions.

#### 2.3.4. Gene Expression Analysis with qRT-PCR

A qRT-PCR reaction was prepared in a 15 µL volume with final concentrations of all ingredients as follows: 1× Maxima SYBR Green qPCR Master Mix (Thermo Fischer Scientific, Waltham, MA, USA), 0.4 µM each primer and 17 ng cDNA. The qRT-PCR was performed in a CFX Opus 96 Real-Time PCR System (Bio-Rad, Hercules, CA, USA) with a thermal program as follows: initial denaturation at 95 °C for 10 min, denaturation at 95 °C for 15 s, annealing/extension at 54–59 °C for 60 s (varied by different primers). The thermal steps 2 and 3 were repeated for 40 cycles. The PCR product sizes were inspected with melting curve analysis and agarose gel electrophoresis. Each cDNA sample was performed in qRT-PCR thrice (technical replications).

Levels of relative expressions of the genes were calculated by the 2^−ΔΔCt^ method [20] in comparison with two housekeeping genes, glyceraldehydes 3 phosphate dehydrogenase (*GADPH*) and elongation factor 1-alpha (*EF-1α*), and the control cDNA derived from the fruit inoculated with water at 0 h. Two-way analysis of variance (ANOVA) was performed, and means were compared by Tukey’s HSD test using the R software version R-4.2.2 [21].

## 3. Results

### 3.1. Fine Mapping and Defense-Related Gene Selection

**Figure 2:**
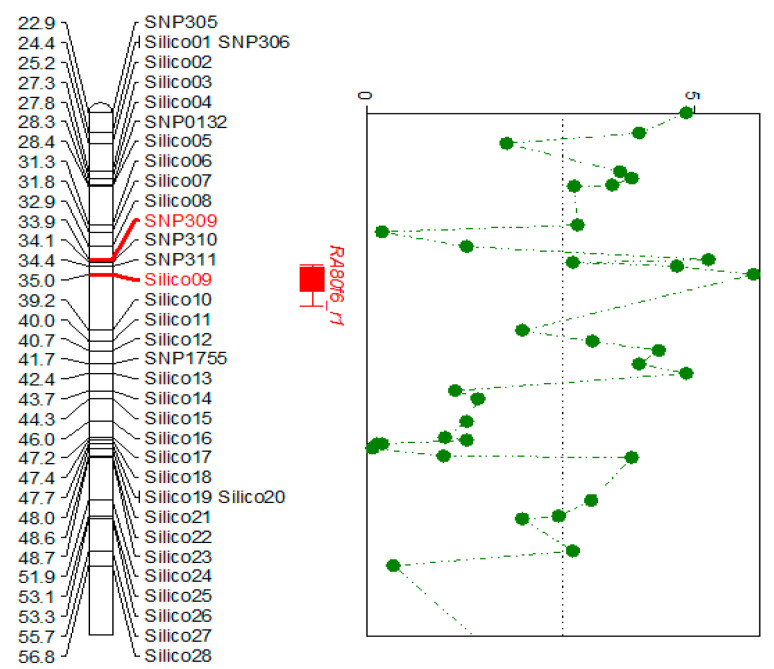
Fine map of the QTL *RA80f6_r1* after the addition of the silico and SNP markers (**left**); LOD values of all the markers in the QTL derived from single marker analysis (**right**), indicating SNP309 and Silico09 had the highest LODs (5.2 and 5.9, respectively).

An additional 80 molecular markers within the QTL *RA80f6_r1* were discovered from the DArTseq genome data of the RIL ‘PBC80’ × ‘CA1316’ population [11]. Of the 80 markers, 69 were silico (insertion-deletion mutants) and 11 were SNPs. Single-marker analysis identified two closely linked markers to the anthracnose resistance loci, Silico09 (34,998,101 bp) and SNP309 (33,876,713 bp), with LOD 5.9 and 5.2, respectively. The physical distance between the Silico09 and SNP309 were 1.12 Mb (Figure 2, Table 2).

Gene searching in the new QTL *RA80f6_r1* region found nine genes, six of which were defense related and three were unknown (Table 3).

### 3.2. Selection of the RILs with Resistance and Susceptibility to Anthracnose

**Figure 3:**
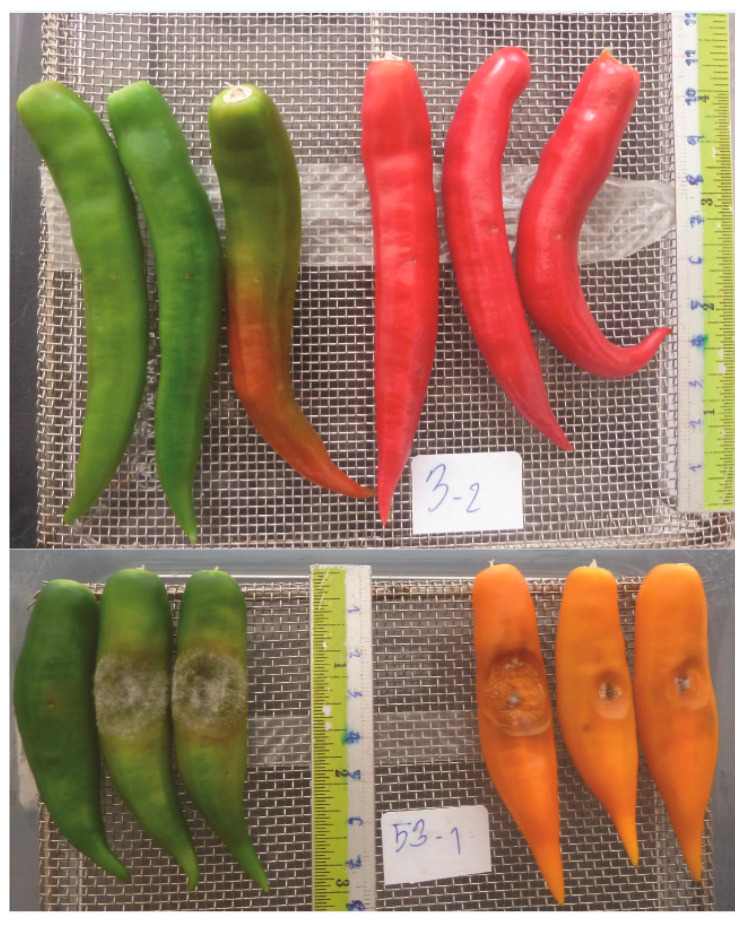
Two selected RILs G1-017130 with R/R (**top**) and G1-017264 with S/S (**bottom**) phenotypes for the gene expression study.

**Figure 4:**
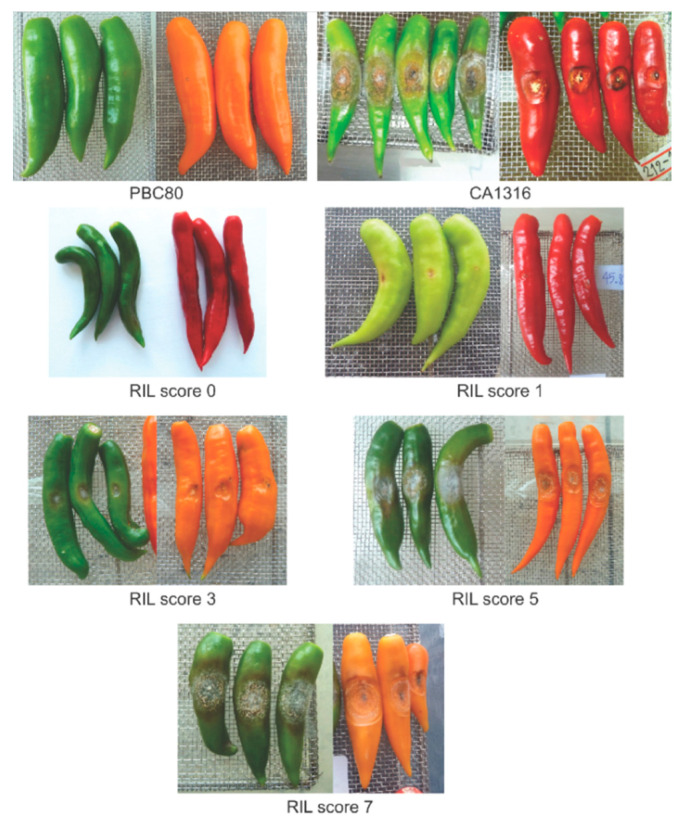
Anthracnose severity scores on mature green and ripe fruit in *Capsicum baccatum* ‘PBC80’ and ‘CA1316’, and in the 29 RILs. The disease scores 0–7 are found in the RILs; score 0 = G1-017425, score 1 = G1-017325 (green) and G1-017473 (ripe), score 3 = G1-017579 (green) and G1-017540 (ripe), score 5 = G1-017541 (green) and G1-017233 (ripe) and score 7 = G1-017267.

Of the 31 RILs, 29 yielded fruit for anthracnose resistance bioassay. Four RILs showed resistance at both fruit stages (R/R), three were resistant at mature green fruit (R/S), eight were resistant at ripe fruit (S/R), and 12 were susceptible at both fruit stages (S/S) (Table 4). Two RILs G1-017130 and G1-017264 were selected with R/R and S/S phenotypes, respectively, (Figure 3, Table 4) for further gene expression study. Anthracnose severity scores of the ‘PBC80’, ‘CA1316’, and the RILs are displayed in Figure 4.

### 3.3. Gene Expression in the Chili Fruit Post Inoculation

**Figure 5:**
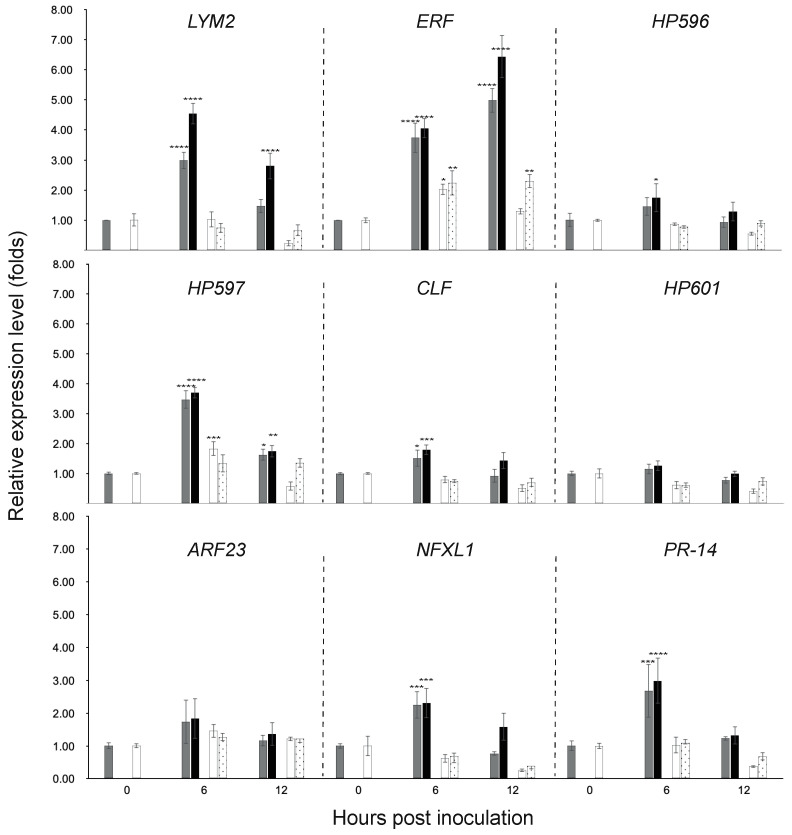
Relative expression levels 0–12 h post fruit inoculation with MJ5 and water of the nine genes in two chili genotypes: R/R + water (grey), R/R + MJ5 (black), S/S + water (white), and S/S + MJ5 (dot). The vertical bars represent standard deviation of the means based on 2-way ANOVA; *, **, *** and **** indicate statistically significant differences compared to the same chili genotype inoculated with water at 0 h (control) at *p* < 0.05, *p* < 0.01, *p* < 0.001 and *p* < 0.0001, respectively, by Tukey’s HSD test.

The gene expression in the fruit after inoculation of the nine genes, including *LYM2*, *ERF*, *HP596*, *HP597*, *CLF*, *HP601*, *ARF23*, *NFXL1*, and *PR-14* were investigated by the qRT-PCR technique. The R/R genotype had six significantly up-regulated genes, i.e., *LYM2*, *ERF*, *HP597*, *CLF*, *NFXL1*, and *PR-14* compared to the control (water inoculation at 0 h; Figure 5). Only *ERF* up-regulated in both chili genotypes; However, the expression was relatively and constantly low in the S/S genotype. Most up-regulated genes reached the highest peak (2.3–4.5 fold) at 6 h and declined at 12 h, except for the *ERF*, which had the highest peak (6.4 fold) at 12 h.

Similar patterns of the gene expressions were also found in the chili fruit inoculated with water. The six genes, i.e., *LYM2*, *ERF*, *HP597*, *CLF*, *NFXL1* and *PR-14*, significantly up-regulated in the R/R genotype and reached the highest peak at 6 h (1.51–3.74 fold) then declined at 12 h, except for the *ERF* that had the highest peak (4.98 fold) at 12 h (Figure 5). The expression levels of these genes in the water control were significantly lower than those in the fruit inoculated with MJ5 (Table 5).

## 4. Discussion

### 4.1. Defense Roles for the Identified Genes

The resistance to anthracnose derived from the ‘PBC80’ has been well documented [8,23]. The defense mechanism is known as a hypersensitive reaction (HR), whereby a form of rapid localized cell death occurs at the infection site to restrict pathogen spread [24]. Plants generally resist a pathogen invasion via two innate immune systems, i.e., cell-surface pattern recognition receptor (PRR)-mediated and intracellular nucleotide-binding leucine-rich repeat receptor (NLR)-mediated immunities [25]. Recent reports have shown that both PRR- and NLR-mediated immunities have mutual roles in HR [26,27,28,29,30], which is defined as pathogen recognition, ion influxes, reactive oxygen species (ROS) burst, lipid peroxidation, transcriptional reprogramming, and cell wall reinforcement [24].

*LYM2* (LysM domain-containing GPI-anchored protein or chitin elicitor-binding protein (*CEBiP*)) is known as PRR. The PRR’s roles in plant defense are pathogen recognition and induction of a plasmodesmata closure, which is the first response by plants to stresses [31]. Once the pathogen is recognized, different signaling cascades in PRR-mediated immunity, i.e., ion influxes, ROS burst, and MAPK cascade are triggered. The recognition roles of *LYM2* as PRR have been reported in rice and *Arabidopsis* [32,33,34]. Moreover, the induction of plasmodesmata closure by *LYM2* was also reported in *Arabidopsis* after being infected by *Botrytis cinerea* [35]. The plasmodesmata closure could prohibit the pathogen spread to the neighboring cells.

*NFXL1* (NF-X1-type zinc finger protein) is a transcription factor identified in *Arabidopsis* [36]. *NFXL1* was reported in *Arabidopsis* for positive regulation of the production of H_2_O_2_, a member of ROS [37]. The accumulation of ROS is one of the earliest defense responses in plants upon pathogen recognition. The ROS’s involvement in the HR through promoting a cell wall reinforcement by forming a cross-linking between glycoproteins [38,39], signaling to stimulate other plant defense responses against pathogens [40,41,42], and participating in programmed cell death (PCD) via the ROS burst process [43,44,45,46].

*CLF* (Histone-lysine N-methyltransferase) has a role in epigenetic regulation of gene expressions through repressive chromatin to silence genes by DNA methylations of H3K27 on the histone H3 [47,48,49,50]. In a stress condition, *CLF* also suppresses the expressions of some genes to properly respond to the environments [51,52,53,54]. Recently, *CLF* was reported to promote a set of defense genes that induced the PCD against a pathogen effector in *Arabidopsis* [55].

*ERF* (Ethylene-responsive transcription factor) is a transcription factor in a subfamily of the APETALA2 (AP2)/ethylene-responsive-element-binding protein (EREBP) in plants [56]. *ERF* plays several different roles during plant development to regulate plant defense responses against abiotic and biotic stresses [57,58,59,60,61,62]. Therefore, *ERF* could promote HR by directly activating the expression of defense-related genes.

*PR-14*, or non-specific lipid-transfer protein 2 (LPT2) is primarily involved in various key processes of plant cytology, i.e., cell wall organization, cell membrane stabilization and signal transduction [63]. In plantdefense, LPT1 and LPT2 have been identified as pathogenesis-related (PR) proteins known as PR-14 [64]. PR proteins are basically against microorganisms. *PR-14* was reported to exhibit antimicrobial activity in mung bean, rice, and wheat against various fungal pathogens, i.e., *Fusarium solani*, *Fusarium oxysporum*, *Pythium aphanidermatum*, *Athelia rolfsii*, *Magnaporthe grisea*, *Rhizoctonia solani*, *Alternaria* sp., *Curvularia lunata*, *Bipolaris oryzae*, *Cylindrocladium scoparium*, *Botrytis cinerea* and *Sarocladium oryzae* [65,66,67], by disrupting the pathogen cell membrane causing loss of membrane integrity [68,69].

### 4.2. Wound Response in Plant Defense Mechanism

Similar to the defense responses to the pathogen invasion, plants with abiotic (wound) stresses activate Ca^2+^ influx, ROS burst, phosphorylation, electrical signaling, the expression of defense-related genes, synthesis of phytohormone, and cell wall reinforcement soon after plant cells are injured [70,71,72]. The inoculation method in the study used a microinjector to wound the pericarp of the chili fruit simultaneously to deliver the inoculum. The fruit inoculated with water also had the same wound-response genes up-regulated as occurred in the ones inoculated with the MJ5 pathogen.

### 4.3. Speculative Roles of LYM2 in HR

HR is a rapid defense mechanism that requires gene receptors to first recognize a pathogen and quickly causes localized cell death in the infected area [12,26]. Several studies in *Arabidopsis* revealed that PRRs and NLRs worked together to trigger HR [26,73,74,75,76]. In the principle, PRRs act by recognizing the pathogen’s elicitor, and subsequently is suppressed by the pathogen’s activities to avoid defense elicitation [77]. NLRs’ role is to support PRRs after being suppressed [25,26]. Therefore, gene receptors appeared to play a key role as an HR trigger in plant defenses.

Recently, *CbAR9*, a *NLR* gene, was reported to be responsible for the HR in the ‘PBC80’ against anthracnose (*Co. truncatum*) [78]. *CbAR9* was found highly expressed at 12 h after fruit inoculation [78]; however, the gene expression was not investigated earlier. *LYM2* has been proven a PRR member [34,35] that showed the highest transcript level (4.53 fold) at the earliest hour (6 h) after fruit inoculation in this study, and thus may have had an important role to induce the HR in ‘PBC80’ chili as well. Based on both studies, *CbAR9* and *LYM2* seemed to involve in the HR in ‘PBC80’ as PRR and NLR receptors.

## 5. Conclusions

A fine map of the QTL *RA80f6_r1* region was achieved by incorporating 80 new markers into the region. Single marker analysis identified two closely linked markers to the anthracnose resistance, Silico09 and SNP309, that were physically 1.12 Mb apart and housing six defense-related and three unknown genes. Five genes, including *LYM2*, *CQW23_09597*, *CLF*, *NFXL1*, and *PR-14* significantly up-regulated in the resistant chili genotype. *LYM2* was the most interesting gene with a receptor function and having the earliest and highest response.

## Figures and Tables

**Table 1:** Sequences of the primers specific to the selected defense genes and housekeeping genes.

Gene ID	Gene Description	Forward/Reverse Primer 5′–3′	Product Size (bp)
CQW23_09568	LysM domain-containing GPI-anchored protein 2 (*LYM2*)	CCCGATCTCTCTTCTCATACAAATGC GGCAGACTTAAGATCCCATCCACAC	133
CQW23_09584	Ethylene-responsive transcription factor (*ERF*)	GGGAAGTTGAGATTGTGAGAAGCA AGGGAGTGAGAATGAGAAGCTGG	172
CQW23_09596	Hypothetical protein (*HP596*)	TCTTTGTCTGAGGTTCCATCGG ACCTTACTACTCTATGCCTTCAAAG	76
CQW23_09597	Hypothetical protein (*HP597*)	CCCAATGAAGAGGATGGCTCTGGT GCAACATCGATTGAACCCCAGAAAC	200
CQW23_09600	Histone-lysine N-methyltransferase (*CLF*)	TTCCTCTGAAGATGCAACTGTG AAGATCCTTCGTCAGATTCTCC	148
CQW23_09601	Hypothetical protein (*HP601*)	TCTTGCTGTGGATCTGTTGCTG TCCTTGCTTTTTGTCTCTGCGG	96
CQW23_09609	Auxin response factor 23 (*ARF23*)	AAAGGTCCGAGCAATCAAAGGG TGCCATCCCTCTCTCTAGAAGC	93
CQW23_09618	NF-X1-type zinc finger protein (*NFXL1*)	TGCTTTTGTGGGAAGAGGCAAG GCAGGGACAACTTCTAGCTGGA	210
CQW23_09644	Non-specific lipid-transfer protein 2 (*PR-14*)	ACAAAGGCAAGGTTTCTGCTCTC GCGATTACATCATCACAACCACCC	72
CQW23_20069	Glyceraldehyde-3-phosphate dehydrogenase (*GAPDH*)	AGACCTTGGAGTTGCAGATGTG TGAGACCGTGACAATGCTAACC	78
CQW23_12274	Elongation factor 1-alpha (*EF-1α*)	TCTCCGACGACAAACCTCAAGC CGCCATTCCTGAATTGTGTGATAGGG	80

**Table 2:** Physical position and LOD values of the markers in relation to the resistance to anthracnose in the QTL *RA80f6_r1* region by single-marker analysis.

Marker	Physical Position (bp)	LOD ^1^	PVE (%) ^2^	Add ^3^
SNP305	22,920,913	4.88	20.87	−0.21
Silico01	24,353,170	4.16	18.07	−0.20
SNP306	24,353,190	4.16	18.07	−0.20
Silico02	25,181,503	2.14	9.78	−0.15
Silico03	27,298,089	3.87	16.92	−0.19
Silico04	27,762,754	4.05	17.67	−0.20
SNP0132	28,348,529	3.75	16.48	−0.19
Silico05	28,350,263	3.17	14.10	−0.18
Silico06	31,275,816	3.22	14.33	−0.18
Silico07	31,813,244	0.24	1.14	−0.05
Silico08	32,891,183	1.53	7.08	−0.13
SNP309	33,876,713	5.22	22.14	−0.22
SNP310	34,133,643	3.15	14.02	−0.17
SNP311	34,378,585	4.74	20.34	−0.21
Silico09	34,998,101	5.91	24.67	−0.23
Silico10	39,171,666	2.38	10.80	−0.15
Silico11	40,015,431	3.45	15.26	−0.18
Silico12	40,678,856	4.46	19.27	−0.20
SNP1755	41,686,959	4.16	18.07	−0.20
Silico13	42,390,824	4.88	20.87	−0.21
Silico14	43,749,964	1.35	6.28	−0.12
Silico15	44,285,168	1.70	7.83	−0.13
Silico16	46,002,881	1.53	7.11	−0.13
Silico17	47,161,782	1.20	5.62	−0.11
Silico18	47,414,359	1.53	7.11	−0.13
Silico19	47,677,887	0.16	0.79	−0.04
Silico20	47,681,951	0.24	1.14	−0.05
Silico21	48,000,095	0.09	0.43	−0.03
Silico22	48,586,639	1.18	5.51	−0.11
Silico23	48,746,637	4.05	17.67	−0.20
Silico24	51,871,096	3.43	15.17	−0.18
Silico25	53,148,783	2.93	13.11	−0.17
Silico26	55,311,209	2.38	10.80	−0.15
Silico27	55,657,165	3.15	14.02	−0.17
Silico28	56,845,351	0.41	1.95	−0.07
SNP7049	65,265,181	2.38	10.80	−0.15
Silico29	65,700,039	2.16	9.86	−0.15
Silico30	65,987,191	0.00	0.04	−0.01
Silico31	68,981,148	2.64	11.91	−0.16
Silico32	71,888,214	2.87	12.87	−0.17
Silico33	72,249,488	3.48	15.36	−0.19
Silico34	75,024,622	3.44	15.20	−0.18
Silico35	78,008,814	3.44	15.20	−0.18
Silico36	78,028,368	3.73	16.38	−0.19
Silico37	78,035,410	0.43	2.05	−0.07
Silico38	78,164,097	2.16	9.86	−0.15
BACSNP_4_63	84,305,804	3.14	14.00	−0.18
Silico39	85,431,446	3.14	14.00	−0.18
Silico40	86,667,602	3.14	14.00	−0.18
Silico41	91,189,255	2.38	10.80	−0.15
Silico42	92,568,227	1.93	8.86	−0.14
Silico43	92,939,149	1.19	5.55	−0.11
Silico44	100,038,292	2.38	10.80	−0.16
Silico45	100,064,499	3.73	16.38	−0.19
Silico46	101,835,880	1.96	8.97	−0.14
Silico47	102,448,025	2.89	12.94	−0.17
Silico48	103,063,217	1.36	6.34	−0.12
Silico49	103,132,977	2.41	10.94	−0.15
Silico50	103,371,531	2.87	12.87	−0.17
Silico51	103,567,675	3.15	14.02	−0.17
Silico52	103,579,898	1.96	8.97	−0.14
Silico53	104,238,202	1.39	6.45	−0.12
Silico54	106,569,759	2.16	9.86	−0.15
Silico55	111,192,822	3.73	16.38	−0.19
BACSNP_4_60	113,019,674	2.16	9.86	−0.15
Silico56	114,140,855	2.41	10.94	−0.15
Silico57	115,808,681	0.80	3.76	−0.09
Silico58	125,855,206	3.18	14.16	−0.17
Silico59	129,409,957	3.15	14.02	−0.17
Silico60	132,674,360	1.59	7.35	−0.13
Silico61	133,136,427	2.38	10.80	−0.15
Silico62	134,969,221	1.77	8.13	−0.13
Silico63	136,379,454	3.44	15.20	−0.18
Silico64	137,027,139	2.87	12.87	−0.17
Silico65	139,918,888	2.00	9.15	−0.14
Silico66	142,635,277	2.89	12.94	−0.17
Silico67	144,664,453	3.74	16.43	−0.19
Silico68	144,808,619	2.64	11.89	−0.17
Silico69	144,901,329	2.93	13.11	−0.17
SNP331	146,776,687	4.74	20.34	−0.21

^1^ LOD: LOD score calculated from single marker analysis. ^2^ PVE: Phenotypic variation explained by the marker. ^3^ Add: Estimated additive effect of the marker.

**Table 3:** Genes with known defense and unknown functions discovered in the new QTL *RA80f6_r1* region.

Gene	Physical Position (bp) ^1^	Gene Function ^2^
*LYM2*	27,166,666–27,169,869	Involved in defense response as chitin-binding protein.
*ERF*	31,226,500–31,227,246	Transcriptional activator that may involve in disease resistance pathways.
*HP596*	33,798,986–33,799,247	Unknown.
*HP597*	34,375,190–34,380,266	Unknown.
*CLF*	34,927,068–34,936,068	Involved in chromosome silencing, histone methylation, regulation of gene expression by genetic imprinting, cell differentiation, etc.
*HP601*	34,997,949–35,013,770	Unknown.
*ARF23*	36,381,979–36,386,588	Transcriptional activator that may involve in disease resistance pathways.
*NFXL1*	36,512,044–36,515,448	Promotes H_2_O_2_ production, defense response to bacterium, response to microbial phytotoxin, response to salt stress, salicylic acid biosynthetic process, etc.
*PR-14*	40,270,316–40,270,594	Transfer lipids across membranes. May play a role in plant defense or in the biosynthesis of cuticle layers.

^1^ Data from genome annotation of *Capsicum baccatum* ‘PBC81’ genome reference [22], National Center for Biotechnology Information “https://www.ncbi.nlm.nih.gov/genome/?term=capsicum+baccatum (accessed on 15 August 2022)”. ^2^ Information from UniProt “https://www.uniprot.org (accessed on 16 August 2022)”.

**Table 4:** Anthracnose severity scores on mature green and ripe fruit of the 29 RILs.

RIL Code	Anthracnose Severity Score (0–9)	
	Mature Green	Ripe
G1-017130	0	0
G1-017135	0	5
G1-017147	5	0
G1-017233	5	5
G1-017257	5	5
G1-017261	5	3
G1-017264	5	5
G1-017267	5	7
G1-017325	1	5
G1-017339	5	0
G1-017396	5	5
G1-017399	0	0
G1-017415	3	0
G1-017416	3	0
G1-017425	0	0
G1-017431	5	1
G1-017433	0	5
G1-017473	0	1
G1-017531	1	1
G1-017539	5	0
G1-017540	5	3
G1-017541	5	5
G1-017544	5	0
G1-017579	3	0
G1-017588	5	5
G1-017593	5	5
G1-017623	5	0
G1-017679	5	5
G1-017845	0	0

**Table 5:** Relative expression levels (means + SD) of the six up-regulated genes in the R/R and S/S chili genotypes at 6 and 12 h post fruit inoculation with water and MJ5.

Genotype + Inoculation	*LYM2*		*ERF*	
	6 h	12 h	6 h	12 h
RR + water	2.99 ± 0.28 ^b^_x_	1.47 ± 0.22 ^c^_y_	3.74 ± 0.51 ^c^_y_	4.98 ± 0.40 ^b^_x_
RR + MJ5	4.53 ± 0.35 ^a^_x_	2.80 ± 0.44 ^b^_y_	4.05 ± 0.33 ^bc^_y_	6.43 ± 0.71 ^a^_x_
SS + water	1.03 ± 0.26 ^cd^_x_	0.24 ± 0.10 ^e^_y_	2.03 ± 0.18 ^de^_x_	1.30 ± 0.10 ^ef^_x_
SS + MJ5	0.75 ± 0.16 ^de^_x_	0.67 ± 0.19 ^de^_x_	2.25 ± 0.42 ^de^_x_	2.30 ± 0.24 ^d^_x_
Genotype + inoculation	*HP597*		*CLF*	
	6 h	12 h	6 h	12 h
RR + water	3.47 ± 0.30 ^a^_x_	1.62 ± 0.20 ^b^_y_	1.52 ± 0.29 ^a^_x_	0.92 ± 0.23 ^cd^_y_
RR + MJ5	3.70 ± 0.19 ^a^_x_	1.75 ± 0.20 ^b^_y_	1.80 ± 0.17 ^a^_x_	1.43 ± 0.28 ^bc^_x_
SS + water	1.83 ± 0.24 ^b^_x_	0.58 ± 0.15 ^d^_y_	0.8 ± 0.11 ^cd^_x_	0.52 ± 0.12 ^d^_x_
SS + MJ5	1.34 ± 0.29 ^bc^_x_	1.36 ± 0.16 ^bc^_x_	0.75 ± 0.06 ^cd^_x_	0.71 ± 0.15 ^cd^_x_
Genotype + inoculation	*NFXL1*		*PR-14*	
	6 h	12 h	6 h	12 h
RR + water	2.25 ± 0.42 ^a^_x_	0.77 ± 0.07 ^c^_y_	2.68 ± 0.82 ^a^_x_	1.23 ± 0.06 ^b^_y_
RR + MJ5	2.31 ± 0.46 ^a^_x_	1.58 ± 0.42 ^ab^_x_	2.98 ± 0.69 ^a^_x_	1.33 ± 0.28 ^b^_y_
SS + water	0.62 ± 0.13 ^c^_x_	0.26 ± 0.04 ^c^_x_	1.02 ± 0.25 ^b^_x_	0.37 ± 0.04 ^b^_x_
SS + MJ5	0.70 ± 0.22 ^c^_x_	0.40 ± 0.10 ^c^_x_	1.09 ± 0.04 ^b^_x_	0.68 ± 0.13 ^b^_x_

Means followed by the same superscript letter (abcdef) within a column or the same subscript letter (xy) within a row of the same gene are not significantly different (*p* > 0.05).

## Data Availability

Not applicable.
